# Co-Fermentation and Genomic Insights into Lactic Acid Bacteria for Enhanced Propionic Acid Production Using a Non-GMO Approach

**DOI:** 10.3390/foods14091573

**Published:** 2025-04-29

**Authors:** Lidia Stasiak-Różańska, Jan Gawor, Kamil Piwowarek, Agata Fabiszewska, Tamara Aleksandrzak-Piekarczyk

**Affiliations:** 1 Department of Food Technology and Assessment, Institute of Food Sciences, Warsaw University of Life Sciences, Nowoursynowska St. 159c, 02-776 Warsaw, Poland; lidia_stasiak_rozanska@sggw.edu.pl; 2 Institute of Biochemistry and Biophysics, Polish Academy of Sciences, Pawinskiego 5a, 02-106 Warsaw, Poland; gaworj@ibb.waw.pl; 3 Department of Food Biotechnology and Microbiology, Institute of Food Sciences, Warsaw University of Life Sciences, Nowoursynowska St. 159c, 02-776 Warsaw, Poland; kamil_piwowarek@sggw.edu.pl; 4 Department of Chemistry, Institute of Food Sciences, Warsaw University of Life Sciences, Nowoursynowska St. 159c, 02-776 Warsaw, Poland; agata_fabiszewska@sggw.edu.pl

**Keywords:** propionic acid, lactic acid bacteria, 1,2-propanediol, co-fermentation, metabolic cooperation, bioprocessing, food preservation

## Abstract

Propionic acid (PA) is an important organic acid with applications in food preservation, feed additives, and bio-based chemical production. While industrial PA is mostly derived from petrochemical processes, sustainable microbial alternatives are gaining attention. In this study, we explored a co-fermentation strategy using lactic acid bacteria (LAB) with complementary metabolic capabilities to enhance PA biosynthesis via the 1,2-propanediol (PDO) pathway. Genome-based screening identified a metabolic division between strains capable of producing PDO (e.g., *Carnobacterium maltaromaticum* IBB3447) and those converting PDO to PA (e.g., *Levilactobacillus brevis* IBB3735). Notably, we discovered that *C. maltaromaticum* IBB3447 is capable of PDO 24 biosynthesis, a function previously undescribed in this species. Phenotypic assays confirmed glycerol metabolism and acid tolerance among strains. In co-culture fermentation trials, the highest PA concentration (6.87 mM) was achieved using simultaneous fermentation in a fructose–sorbitol–glucose (FRC-SOR-GLC) medium, accompanied by prior PDO accumulation (up to 13.13 mM). No single strain produced PA independently, confirming that metabolic cooperation is required. These findings reveal a novel LAB-based bioprocess for sustainable PA and PDO production, using cross-feeding interactions and the valorization of industrial waste streams. The study supports future optimization and scale-up for circular bioeconomy applications.

## 1. Introduction

Propionic acid (PA) is an industrially significant short-chain fatty acid widely used as a preservative in food and feed, as well as a precursor for bioplastics, pharmaceuticals, and other specialty chemicals. The global demand for PA was 470,000 tons in 2019 and is projected to reach 550,000 tons by 2026 [1]. Although PA is primarily synthesized via petrochemical routes [2], growing concerns regarding environmental sustainability have led to increased interest in microbial PA production as a bio-based alternative. Traditionally, *Propionibacterium* spp. have been considered the primary microbial producers of PA, but their production is limited by acid accumulation and pH-associated growth inhibition [3,4]. Alternative microbial pathways for PA biosynthesis, including the acrylate pathway in *Clostridium propionicum* and *Megasphaera elsdenii*, as well as the 1,2-propanediol (PDO) pathway in *Salmonella enterica*, also present challenges such as toxicity of metabolic intermediates or virulence of the producing organisms [5,6,7].

Lactic acid bacteria (LAB) are Gram-positive, generally recognized as safe (GRAS) microorganisms that are widely used in food fermentation and probiotics. While they are primarily known for lactic acid production, some LAB species can produce other low-molecular-weight organic compounds, including acetic acid, ethanol, and small amounts of PA, particularly when fermenting fucose or rhamnose [8,9]. Previous studies suggest that co-fermentation strategies involving *Lentilactobacillus buchneri* and *Lactobacillus diolivorans* may enhance PA biosynthesis due to metabolic complementarity, by which *L. buchneri* converts lactate to PDO, which is subsequently transformed into PA by *L. diolivorans*. This metabolic synergy has been observed in silage fermentation, for which LAB contribute to PA production, inhibiting molds and improving silage stability without requiring additional preservatives [10,11]. However, the enzymatic pathways and genetic determinants underlying PA biosynthesis in LAB remain poorly characterized, necessitating further research [7].

PDO biosynthesis follows three major microbial pathways: (i) the deoxyhexose pathway, found in *Klebsiella* spp. and *Clostridium* spp., where L-rhamnose or L-fucose are metabolized to PDO; (ii) the methylglyoxal pathway, found in *Clostridium sphenoides*, *Escherichia coli*, and *Proteus vulgaris*, which converts glycerone phosphate (dihydroxyacetone phosphate, DHAP) to PDO via methylglyoxal intermediates; and (iii) the lactate pathway, identified in some LAB species, including *Levilactobacillus brevis* and *L. buchneri* [12,13]. Among these, the lactate pathway is particularly attractive for industrial applications, as it avoids toxic intermediates (such as methylglyoxal in the methylglyoxal pathway) and does not require expensive carbon sources (e.g., fucose or rhamnose in the deoxyhexose pathway) [14]. In this pathway, lactic acid is converted into lactoyl-CoA, reduced to lactaldehyde, and subsequently transformed into PDO, often under acidic conditions [13,15,16].

Some microorganisms possess the PDO-to-PA conversion pathway, where PDO is first dehydrated to propionaldehyde by propanediol dehydratase, followed by oxidation to propionyl-CoA and subsequent PA formation via multiple enzymatic routes [7,17]. This biochemical potential of certain LAB to metabolize PDO into PA makes them promising candidates for alternative PA production strategies.

Beyond its industrial applications, PA is a highly effective preservative, widely used in bakery products, silage fermentation, and food safety applications. As a weak organic acid, it disrupts cell membranes of bacteria and fungi, inhibits metabolic reactions, and induces intracellular acid stress, making it highly effective for controlling spoilage and extending product shelf life [18,19]. The increasing demand for natural, bio-based preservatives is expected to drive market growth, particularly in clean-label food production, animal nutrition, and sustainable biotechnology [20,21]. Emerging markets in Asia, Latin America, and Africa present significant opportunities for the expansion of microbial PA production, further emphasizing the need for efficient, cost-effective, and sustainable biotechnological production methods [22,23]. PA biosynthesis is also highly relevant in cheese production, in which it contributes to flavor development and eye formation in Swiss-type cheeses, imparting characteristic nutty and slightly sweet notes [24]. The potential to design co-fermentation strategies leveraging LAB metabolic complementarities could pave the way for the development of novel starter cultures that not only drive lactic acid fermentation but also facilitate PA biosynthesis, thereby enhancing cheese quality and functionality.

Given the growing demand for natural preservatives and sustainable chemical production, LAB represent a promising platform for PA biosynthesis. This study investigates LAB-driven PA production through co-fermentation strategies, integrating metabolic and genomic analyses to elucidate the underlying pathways. While individual LAB strains lack complete PA biosynthetic pathways, metabolic cooperation between complementary strains enables PA synthesis, offering a viable alternative to conventional production methods. The process uses affordable and renewable carbon sources such as glycerol (a low-cost byproduct of biodiesel production) and sorbitol (a sugar alcohol available from starch-processing industries), enhancing economic feasibility and supporting circular bioeconomy applications. By addressing critical knowledge gaps, this research aims to explore the feasibility of harnessing LAB for PA biosynthesis, with potential applications in food preservation, animal nutrition, and industrial biotechnology [25,26].

In this study, we demonstrate that LAB can produce PDO and propionic acid PA through metabolic cooperation. Genome sequencing and bioinformatics analyses identified key PDO pathway genes, revealing *C. maltaromaticum* IBB3447 as a novel PDO producer, capable of utilizing glycerol, an industrial byproduct, for sustainable PDO synthesis. In co-fermentation, this strain contributed to PA biosynthesis, highlighting its potential for circular bioeconomy applications. Controlled co-fermentation trials confirmed that LAB consortia optimize PA production, with simultaneous fermentation yielding the highest PA concentrations due to enhanced PDO accumulation. These findings establish LAB as viable platforms for bio-based PA and PDO production, offering new opportunities for sustainable bioprocessing and waste valorization.

## 2. Materials and Methods

### 2.1. In Silico Analysis of LAB Genomes

To identify LAB strains with the potential for PA biosynthesis, we performed an in silico screening of publicly available bacterial genomes. Genomic data were retrieved from databases including the National Center for Biotechnology Information (NCBI), GenBank (https://www.ncbi.nlm.nih.gov/genbank/, accessed on 31 January 2025) [27], and UniProt (https://www.uniprot.org/) [28] (accessed on 31 January 2025). These databases provide comprehensive genomic information, including complete and draft genome sequences, as well as annotations of protein-coding genes. The analysis focused on identifying genes encoding enzymes involved in the three known PA biosynthesis pathways: the succinate pathway, the acrylate pathway, and the PDO pathways. We employed the Kyoto Encyclopedia of Genes and Genomes (KEGG) database (https://www.genome.jp/kegg/, accessed on 31 January 2025) [29] and its associated tools, specifically the KEGG Mapper (https://www.genome.jp/kegg/tool/map_pathway.html, accessed on 31 January 2025) and KEGG Pathway tools (https://www.genome.jp/kegg/pathway.html) (accessed on 31 January 2025), to identify homologous genes and reconstruct metabolic pathways. Additionally, we utilized BLAST (Basic Local Alignment Search Tool) (https://blast.ncbi.nlm.nih.gov/Blast.cgi, accessed on 31 January 2025) for sequence similarity searches and HMMER (http://hmmer.org/) [30] (accessed on 31 January 2025) for identifying protein domains characteristic of enzymes involved in PA biosynthesis. For pathway reconstruction, we utilized the KEGG Mapper tool, which allowed us to visualize and compare metabolic pathways across different LAB strains. This tool facilitated the identification of complete or partial PA biosynthesis pathways within the selected genomes. Furthermore, we employed the Pathway Tools software (https://pathwaytools.com/, accessed on 31 January 2025) [31], specifically the PathoLogic component, to build pathway/genome databases (PGDBs) and reconstruct metabolic networks for selected LAB strains, providing a comprehensive overview of their metabolic capabilities (accessed on 31 January 2025).

### 2.2. Isolation of Strains

The bacterial strains used in this study were isolated from various natural sources and deposited in microbial collections. *C. maltaromaticum* IBB3447 was isolated from bovine milk, while *L. brevis* IBB3734 and IBB3735 were obtained from different sources of sauerkraut and are stored in the COLIBB collection at the Institute of Biochemistry and Biophysics, Polish Academy of Sciences (IBB PAS, Warsaw, Poland). Additionally, *L. buchneri* A KKP 2047p, a strain isolated from corn silage, was obtained from the Collection of Industrial Microorganisms at the Prof. Wacław Dąbrowski Institute of Agricultural and Food Biotechnology, State Research Institute (IBPRS-PIB, Warsaw, Poland). The *L. buchneri* A KKP 2047p strain was characterized by its ability to synthesize PDO, while lacking the ability to metabolize PDO for bacterial growth [32,33,34]. This strain, along with the formulation of a bacterial preparation designed for preserving high-starch plants, has been patented [Pat.219782, EP2785826B1, US 9370199].

### 2.3. Culture Conditions

For monoculture cultivation, the base medium consisted of either de Man, Rogosa, and Sharpe (MRS) broth or M17 broth, supplemented with 0.002% vitamin B12, 3% sugar, and 1% inoculum of the respective strain. Overnight cultures were grown to late exponential phase (OD_600_ ≈ 1.5) and used to inoculate fresh media at 1% (*v*/*v*) final concentration for all fermentation experiments, ensuring consistent starting biomass across all assays. For co-fermentation experiments, the medium was supplemented with the following: 3% sugar (mannose, fructose, glycerol, glucose, rhamnose, sorbose, or sorbitol; Merck, Darmstadt, Germany), 1% inoculum of the second strain, 0.002% vitamin B12, 2% of a 5× concentrated MRS broth, and 0.8% of PDO when needed. Cultures were incubated while stationary at 30 °C under either aerobic or relatively anaerobic conditions, depending on the strain used. To monitor the dynamics of metabolite synthesis, supernatant samples were collected at 3, 4, 7, 14, and 21 days. All sugars, broths, and supplements used in this study were sourced from Chempur (Piekary Śląskie, Poland), Pol-Aura (Warsaw, Poland), Thermo Fisher Scientific (Waltham, MA, USA), and Merck (Darmstadt, Germany).

The bacterial co-culture was conducted in two approaches: stepwise fermentation and simultaneous fermentation. For stepwise fermentation, a strain from Group I (strains capable of producing PDO) was initially cultivated as a monoculture using selected carbon sources. After a predetermined incubation period (ranging from 96 h to 14 days, based on previous monoculture experiments), the levels PA and PDO production were assessed. Subsequently, an additional carbon source, previously identified as optimal for PA production, was introduced into the culture, followed by the inoculation of a strain from Group II (strains capable of converting PDO to PA). The co-culture was then incubated for 96 h, after which the levels of PA and PDO were measured. For simultaneous fermentation, strains from Group I and Group II were co-cultured from the beginning, using their respective carbon sources. The incubation time was determined based on previous monoculture experiments. Following fermentation, the production levels of PA and PDO were analyzed.

### 2.4. Whole-Genome Sequencing (WGS)

Short-read bacterial genome sequencing was performed using the MiSeq instrument (Illumina Inc., San Diego, CA, USA). Genomic DNA was purified using the SDS/Phenol method [35]. DNA quality control was performed by measuring the absorbance at 260/230 using PicoDrop, concentration was determined using a Qubit fluorimeter (Thermo Fisher Scientific, Waltham, MA, USA), and DNA integrity was analyzed by 0.8% agarose gel electrophoresis. DNA libraries were constructed using an NEB Ultra II FS kit (NEB, Ipswich, MA, USA), followed by paired-end 300 basepair sequencing (aiming for at least 50x genome coverage). Sequence quality metrics were assessed using FASTQC (http://www.bioinformatics.babraham.ac.uk/projects/fastqc/, accessed on 31 January 2025) [36]. Raw sequencing reads were trimmed for quality and residual library adaptors were removed using fastp v.0.23.4 [37] (https://www.biorxiv.org/content/early/2018/04/09/274100, accessed on 31 January 2025). Cleaned short reads were checked for contamination using Kraken2 v.2.0.8 (https://ccb.jhu.edu/software/kraken2/). Illumina reads were then assembled into contigs using SPAdes v.3.15.5 (https://github.com/ablab/spades, accessed on 31 January 2025) and Unicycler v.0.4.8 pipeline (https://github.com/rrwick/Unicycler, accessed on 31 January 2025) to obtain high-quality draft genomes of each bacterial isolate. The final assemblies were evaluated using Quast v.4.5 (http://quast.sourceforge.net/quast, accessed on 31 January 2025) software. Coding sequence (CDS) prediction and gene annotation were performed using the NCBI Prokaryotic Genome Annotation Pipeline (v.6.6) [38]. Obtained genomes were searched for PA genes and biosynthesis pathways by the same bioinformatic tools as described in the section “In silico analysis of LAB genomes”.

### 2.5. BIOLOG Phenotypic Analysis

The BIOLOG Phenotype MicroArray™ (PM) system (Biolog, Hayward, CA, USA) was used to assess the metabolic activity of bacterial strains under various conditions. The procedure was generally performed according to Kosiorek et al. [39], with modifications tailored to the specific requirements of the tested strains. Bacterial colonies were scraped from MRS agar plates and resuspended in IF-0a fluid (Biolog, Hayward, CA, USA) to a final transmittance of 65–81%, depending on the strain. The suspension was supplemented with growth additives and Biolog redox tetrazolium dye (Dye Mix B or G, Biolog, Hayward, CA, USA), following standard BIOLOG protocols for *Lactobacillus* species. The prepared bacterial suspensions were inoculated into PM1 and PM2 plates, which assess carbon source utilization, and incubated in an OmniLog reader for up to 72–96 h at 30 °C. For metabolic kinetics assessment, the OmniLog system recorded optical density changes every 15 min, with results expressed in OmniLog arbitrary units (OAUs). The area under the curve (AUC) of metabolic activity was calculated and averaged for comparative analysis. The collected kinetic data were processed and analyzed using OmniLog Data File Converter and OmniLog PM software (v.1.20.02).

### 2.6. Antagonistic Activity Assay of Bacterial Strains

Several experimental conditions were tested in quadruplicate: glucose, fructose, rhamnose, and mannose under aerobic, anaerobic, and microaerophilic conditions. The double-layer agar plate method was used to evaluate antimicrobial activity. Petri dishes containing solid growth medium were first overlaid with a soft agar layer (0.7% agar) inoculated with 100 µL of the indicator strain (*C. maltaromaticum* IBB3447, *L. buchneri* A KKP 2047p, *L. brevis* IBB3734, or *L. brevis* IBB3735) to ensure uniform distribution. After solidification, 5 µL of the culture of the producer strain (tested in all pairwise combinations) was spotted onto the surface of the soft agar layer. The plates were incubated at 30 °C for 48 h under the designated oxygen conditions. Following incubation, antimicrobial activity was assessed by measuring the presence or absence of a clear inhibition zone surrounding the producer strain’s growth area. The absence of a clearance zone indicated no inhibitory effect against the indicator strain.

### 2.7. Gas Chromatography (GC) Analysis

The concentrations of PA and PDO were determined using gas chromatography with a flame ionization detector (GC-FID) (Trace 1300, Thermo Fisher Scientific, Waltham, MA, USA). Chromatographic separation was performed on a ZB-WAXplus column (30 m × 0.25 mm × 0.25 μm). In these assays, samples from at least two independent cultures were analyzed. For PA analysis, samples were centrifuged at 10,000 rpm for 10 min (Centrifuge 5804R, Eppendorf). One milliliter (1 mL) of the supernatant was transferred to a screw-cap reaction tube, followed by the addition of 2 mL of the extraction mixture (hexane:diethyl ether, 1:1, *v*/*v*). The samples were shaken to extract PA, and the internal standard (undecanoic acid, Sigma-Aldrich, St. Louis, MO, USA) was added. After extraction, the samples were transferred to GC vials. The temperature program for PA analysis was as follows: initial temperature: 40 °C (2 min) and ramp: 5 °C/min to 180 °C (hold for 15 min). The injector and FID detector temperatures were set at 230 °C and 260 °C, respectively. The injected sample volume was 2 μL, and nitrogen was used as the carrier gas at a column flow rate of 1.6 mL/min. Qualitative analysis of PA was performed by comparing the retention times of the samples with an analytical standard of PA, while quantitative calculations were performed using the internal standard method. For PDO determination, sample preparation was conducted according to Egoburo et al. [40]. The temperature program for PDO analysis was as follows: initial temperature: 80 °C (1 min) and ramp: 10 °C/min to 260 °C (hold for 2 min). The injector and detector temperatures were set at 240 °C and 350 °C, respectively. The injected sample volume was 2 μL, with nitrogen as the carrier gas at a flow rate of 1.5 mL/min. Qualitative analysis of PDO was based on retention time comparison with an analytical standard of PDO, while quantitative calculations were performed using a standard curve.

## 3. Results

### 3.1. Genomic Screening and Identification of LAB with Potential for PDO and PA Synthesis

KEGG-based analysis of propanoate metabolism revealed differences in the prevalence of enzymes converting glycerone-P to PA among bacterial species. Enzymes involved in glycerone-P-to-PDO conversion (methylglyoxal pathway) are more widely distributed than those responsible for subsequent PDO transformation into propionyl-CoA (Supplementary Figure S1), suggesting that the initial pathway segment is more conserved and frequently utilized. For this study, bacteria possessing the complete enzymatic methylglyoxal pathway for PDO biosynthesis were classified as Group I, while those lacking this capability but with the genetic potential to metabolize PDO into propionyl-CoA were designated as Group II. The methylglyoxal synthesis pathway begins with methylglyoxal synthase (EC 4.2.3.3), which is highly represented among genome-sequenced bacterial species and converts glycerone-P into methylglyoxal. Methylglyoxal is then processed by glycerol dehydrogenase (EC 1.1.1.6) or methylglyoxal reductase (EC 1.1.1.283) to L-lactaldehyde, which is subsequently reduced to PDO by lactaldehyde reductase (EC 1.1.1.77), an enzyme less frequently encoded in bacterial genomes. Alternative steps involve alcohol dehydrogenase YqhD (EC 1.1.-.-) or methylglyoxal reductase YdjG (EC 1.1.1.-), which convert methylglyoxal to hydroxyacetone, and glycerol dehydrogenase (EC 1.1.1.6), catalyzing the reduction of hydroxyacetone to PDO. The next step in the glycerone-P-to-PA conversion is catalyzed by PDO dehydratase (EC 4.2.1.28), which dehydrates PDO to propanal. This is followed by propanal dehydrogenase (EC 1.2.1.87), which oxidizes propanal to propionyl-CoA. Genes encoding these two enzymes are underrepresented in bacterial genomes. The final step, the conversion of propionyl-CoA to PA, is catalyzed by various enzymes encoded by genes with a broad distribution across bacterial taxa (Supplementary Figure S1).

In silico genome screening at the GenBank database revealed that LAB lack a complete biosynthetic glycerone-P-to-PA pathway, and the majority of them do not even carry genes that encode enzymes for the entire methylglyoxal or PDO-PA pathways. However, some species may compensate for this shortage through metabolic cooperation, understood as a division of metabolic tasks between strains with complementary enzymatic capabilities. In this model, Group I bacteria synthesize PDO from various carbon sources via the methylglyoxal pathway and thereby act as PDO donors, while Group II bacteria, which lack this pathway, can uptake and convert PDO to PA. Group I includes certain species or strains from the genera *Carnobacterium*, *Ligilactobacillus*, *Aerococcus*, *Jeotgalibaca*, *Dolosigranulum,* and certain *Enterococcus* species, while Group II includes *Limosilactobacillus*, *Levilactobacillus*, *Loigolactobacillus*, *Secundilactobacillus*, *Pediococcus*, *Furfurilactobacillus*, *Companilactobacillus*, and *Latilactobacillus*. For example, *Lentilactobacillus buchneri* exhibited only an incomplete methylglyoxal pathway, containing glycerol dehydrogenase (EC 1.1.1.6), but not the full set of enzymes required for PDO synthesis. This metabolic complementarity enables a cross-feeding mechanism: Group I bacteria contribute to PDO production, while Group II bacteria utilize the PDO for PA synthesis, forming a cooperative two-step pathway distributed across microbial partners. These observations led us to hypothesize that LAB strains from different functional groups may complement each other’s metabolic capabilities and jointly enable PA biosynthesis in co-culture systems.

This in silico analysis identified LAB strains with complementary metabolic capabilities that together could support PA biosynthesis. The observed metabolic division between Group I and Group II strains lays the foundation for designing co-fermentation strategies based on cross-feeding interactions. These findings provide a rationale for the experimental validation of selected strain combinations and highlight the ecological and biotechnological relevance of distributed biosynthetic pathways in LAB.

### 3.2. Genome Sequencing and Identification of PA Biosynthesis Genes

To further investigate the potential for PA biosynthesis, selected strains from our in-house and IBPRS-PIB collection were subjected to whole-genome sequencing. These included representatives from both functional groups: *L. buchneri* A KKP 2047p and *C. maltaromaticum* IBB3447 (Group I), as well as *L. brevis* IBB3734 and *L. brevis* IBB3735 (Group II). Genome assembly statistics were collected in Table S1. Metabolic pathway analyses confirmed the presence of genes required for glycerone-P-to-PDO conversion via the methylglyoxal pathway in *C. maltaromaticum* IBB3447 and for PDO-to-PA conversion in both *L. brevis* strains. Additionally, genomic analysis of the PDO pathways in *L. buchneri* identified only an incomplete methylglyoxal pathway, containing glycerol dehydrogenase (EC 1.1.1.6), further supporting in silico findings from publicly available genomes in GenBank. In this strain, genes encoding enzymes for the complete lactate pathway (propionate CoA-transferase [EC 2.8.3.1] and propanal dehydrogenase [EC 1.2.1.87]) were also not identified, further questioning the route for PDO biosynthesis in *L. buchneri* A KKP 2047p. These results suggest that while no single LAB strain possesses a complete PA biosynthesis pathway, metabolic cooperation between strains from Groups I and II may facilitate PA production, warranting further investigation through co-fermentation approaches.

### 3.3. Phenotypic Characterization of LAB Strains

Phenotypic characterization using Biolog phenotype microarrays showed that most tested LAB strains could efficiently metabolize various carbon sources relevant to PA biosynthesis, including glucose, fructose, rhamnose, and sorbitol. Notably, *C. maltaromaticum* IBB3447 demonstrated the ability to metabolize glycerol, a potential substrate from industrial waste streams. Acid tolerance assays indicated that all strains could survive at pH of 3–3.5; however, none of them could directly utilize PA or PDO as a primary carbon source, suggesting that PA production in LAB occurs only as a secondary metabolic process.

### 3.4. Influence of Carbon Source and Oxygen Availability on Antagonistic Interactions

To evaluate the feasibility of co-fermentation strategies, antagonistic interactions among *C. maltaromaticum* IBB3447, *L. buchneri* A KKP 2047p, *L. brevis* IBB3734, and *L. brevis* IBB3735 were examined under aerobic, anaerobic, and microaerophilic conditions in different carbon sources such as glucose, fructose, rhamnose, or mannose. The results indicate that antagonistic bacterial activities were the same under each of the tested oxygen conditions. *C. maltaromaticum* IBB3447 exhibited strong inhibition against *L. buchneri* A KKP 2047p and both *L. brevis* strains in mannose, while moderate inhibition was observed against *L. brevis* IBB3735 in fructose. Some antimicrobial activity was also detected for *L. buchneri* A KKP 2047p and *L. brevis* IBB3735, both of which displayed slight antagonistic activity in mannose under aerobic conditions. In contrast, *L. brevis* IBB3734 did not exhibit any inhibitory activity against the tested indicator strains. Glucose did not induce antagonistic activity against any of the strains, similar to rhamnose (Table 1). These findings indicate that antagonistic activity is strain-specific and influenced by the carbon source but not oxygen availability. The observed inhibition patterns suggest potential compatibility or incompatibility in co-fermentation strategies involving these strains.

### 3.5. PA and PDO Biosynthesis in Monocultures

Initial flask experiments with monocultures of four lactic acid bacteria (LAB) strains over a period of 3 to 14 days demonstrated PDO accumulation in Group I strains, whereas Group II strains exhibited only limited PDO synthesis (Figures S3–S5). Among the tested strains, only *C. maltaromaticum* IBB 3447 synthesized PDO across all tested carbon sources, including mannose, fructose, glycerol, glucose, rhamnose, sorbose, sorbitol, and fucose (Figure S3). The highest relative PDO content was observed in M17 medium supplemented with sorbitol after 96 h, which served as a reference for other carbon sources. Glycerol and rhamnose also supported high PDO production, peaking at 90% and 93%, respectively, after 96 h. In contrast, fucose consistently resulted in the lowest PDO levels, remaining below 5% of the maximum obtained in sorbitol medium throughout the experiment. Fructose exhibited a progressive increase over time, reaching 67.3% at 14 days, while mannose and glucose peaked earlier, at 47% and 64%, respectively. Sorbose led to moderate PDO production, peaking at 74% after 96 h, but became undetectable by day 14 (Figure S3). These findings indicate that sorbitol is the most effective carbon source for PDO production by *C. maltaromaticum* IBB 3447.

The analysis of PDO production by *L. buchneri* A KKP 2047 revealed significant variations depending on the incubation time and carbon source (Figure S4). This strain failed to produce PDO in the presence of most carbon sources, including glycerol, rhamnose, L-sorbose, D-sorbitol, and L-fucose. In a mannose-based medium, the PDO content decreased during the first seven days, followed by a substantial increase on day 14. Fructose initially remained at very low levels but sharply increased by day 14, making it the primary sugar associated with the highest PDO concentration at this time point (Figure S4). In contrast, in a glucose-based medium, PDO was largely depleted after 96 h and remained undetectable until there was a slight increase (5%) on day 14.

Monoculture experiments with *L. brevis* IBB3734 and *L. brevis* IBB3735 revealed a complete lack of PDO production, regardless of the carbon source used. Additionally, none of the tested strains were capable of producing PA in the culture medium unless PDO was supplemented. Following the addition of PDO, PA synthesis was observed only in the presence of *L. brevis* strains, which produced PA exclusively in media containing glucose and fructose, with the highest production rates occurring between days 4 and 7 (Figure S5). No PA biosynthesis was detected in other strains, even after PDO supplementation.

### 3.6. Co-Fermentation for PA Biosynthesis in LAB and Bioreactor Co-Fermentation Trials

Based on the results assessing the effect of carbon sources on bacterial interactions, PDO and PA production, and optimized culture duration, we evaluated co-fermentation approaches using Group I (*C. maltaromaticum* IBB3447 and *L. buchneri* A KKP 2047p) and Group II (*L. brevis* IBB3735 and IBB3734) LAB strains. To determine the most efficient method for cooperative PA biosynthesis, two strategies were compared: stepwise fermentation and simultaneous fermentation.

In the stepwise fermentation approach, *C. maltaromaticum* IBB3447 was cultivated in glycerol, sorbitol, or rhamnose for 96 h, producing PDO concentrations ranging from 1.20 to 1.52 mM in glycerol, 1.39 to 1.52 mM in sorbitol, and 1.30 to 1.41 mM in rhamnose, while PA was not detected. In contrast, *L. buchneri* A KKP 2047p grown in fructose for 14 days exhibited higher PDO production (3.10 to 3.65 mM) but similarly lacked PA synthesis. When *L. brevis* IBB3734 or IBB3735 were added to the respective *C. maltaromaticum* or *L. buchneri* monocultures and incubated in glucose for 96 h, PDO production was largely depleted, while PA concentrations increased, ranging from 0.17 to 0.73 mM. Notably, in co-cultures of *L. brevis* with *L. buchneri*, PDO remained at relatively high levels (1.57 to 1.81 mM), indicating incomplete conversion to PA, despite PA concentrations reaching 1.45 to 3.31 mM. These findings suggest that *L. buchneri* exhibits a higher intrinsic ability for PDO production than *C. maltaromaticum* and that *L. brevis* strains play a key role in converting PDO to PA, albeit with occasional accumulation of PDO due to incomplete metabolic conversion (Table 2).

In the simultaneous fermentation approach, distinct patterns of PDO and PA biosynthesis emerged depending on the strain combination and carbon source. When *C. maltaromaticum* IBB3447 was co-cultured with *L. brevis* IBB3735, PA biosynthesis was strongly promoted in the presence of glucose and rhamnose. Here, PDO production declined over time, while PA steadily increased, suggesting an active metabolic conversion process. In the GLC-SOR combination, PDO peaked at 0.92 mM at 72 h, declined to 0.31 mM at 7 days, and was almost absent by day 14, whereas PA progressively increased to 0.92 mM at 14 days. A similar trend was observed in GLC-RHA, in which PDO started at 0.80 mM at 72 h and dropped to 0.11 mM at 7 days, and PA levels increased to 0.78 mM at 14 days, confirming the ability of *L. brevis* IBB3735 to convert PDO into PA. In contrast, *C. maltaromaticum* IBB3447 co-cultured with *L. buchneri* A KKP 2047p exhibited no PA biosynthesis initially, but upon the addition of *L. brevis* IBB3735, PA production increased significantly. In fructose and sorbitol media, *C. maltaromaticum* IBB3447 and *L. buchneri* A KKP 2047p produced high levels of PDO (up to 13.13 mM at 14 days in FRC-RHA), yet PA remained undetectable. However, upon the introduction of *L. brevis* IBB3735, PA production surged, with the highest yield recorded in FRC-SOR-GLC, reaching 6.87 mM on day 14. Similarly, in FRC-SOR, PA levels increased to 5.94 mM at 14 days, demonstrating the pivotal role of *L. brevis* in converting PDO into PA. The highest PDO production was observed in FRC-RHA, reaching 13.13 mM at 14 days, while the highest PA production was achieved in FRC-SOR-GLC, reaching 6.87 mM at 14 days. These results underscore the importance of carbon source selection and strain synergy, where *L. brevis* IBB3735 enhances PA biosynthesis by efficiently utilizing PDO, a function not observed in *C. maltaromaticum* IBB3447 or *L. buchneri* A KKP 2047p alone (Table 3).

Overall, the simultaneous fermentation approach demonstrated higher PA production efficiency, but this could be largely influenced by the initially elevated PDO levels observed when both *C. maltaromaticum* IBB3447 and *L. buchneri* A KKP 2047p were co-cultured. Unlike the stepwise approach, in which only a single Group I strain was used at a time, the simultaneous presence of two PDO-producing strains resulted in unexpectedly high PDO accumulation before the introduction of *L. brevis* IBB3735. This created an optimal metabolic environment for PDO conversion into PA, ultimately leading to the highest PA yields, reaching 6.87 mM. While the stepwise fermentation method allowed for controlled metabolic interactions, its overall efficiency was limited by lower initial PDO levels. In contrast, the simultaneous fermentation strategy, by using the synergistic PDO production of two Group I strains, provided a substrate-rich environment for PA biosynthesis, making it a more effective and scalable biotechnological process for PA production.

## 4. Discussion

This study investigated the potential of lactic acid bacteria (LAB) for propionic acid (PA) biosynthesis through co-fermentation strategies using the 1,2-propanediol (PDO) pathway. The results demonstrate that LAB strains, although individually lacking complete PA biosynthetic pathways, can metabolically cooperate to achieve PA production. Specifically, *C. maltaromaticum* IBB3447 and *L. buchneri* A KKP 2047p synthesized PDO, while *L. brevis* IBB3734 and IBB3735 converted PDO into PA, confirming their complementary metabolic roles.

The microbial production of PDO from rhamnose or fucose under anaerobic conditions has been deemed commercially unfeasible due to high substrate costs and low yield [16]. Therefore, this study focused on identifying inexpensive and readily available carbon sources, such as glucose and fructose, for co-fermentation-based PDO and PA production. The results indicate that *L. brevis* produced PA only in the presence of fructose and glucose, with maximum concentrations (~1 mM) observed at 96 h, followed by a gradual decline. Notably, no PA production was detected in monocultures without PDO supplementation, confirming that PA biosynthesis in *L. brevis* depends on the presence of PDO, supporting the functional PDO-to-PA conversion pathway identified in this strain.

Among the tested LAB candidates, *L. brevis* exhibited the highest PDO-to-PA conversion efficiency. To further enhance PA biosynthesis, bioreactor co-fermentation trials were performed using selected LAB pairs. Co-cultures of *L. buchneri* + *L. brevis* and *C. maltaromaticum* + *L. brevis* yielded the highest PA production, reaching 6.87 mM without external PDO supplementation, highlighting the effectiveness of metabolic exchange between LAB strains in a controlled bioreactor system. These results indicate that the simultaneous co-culture of PDO-producing and PA-converting LAB strains under optimized carbon source conditions provides an efficient metabolic environment for propionic acid biosynthesis. The lack of external PDO supplementation further emphasizes the strength of the in situ substrate generation and utilization within the consortia. This finding confirms the hypothesis that LAB strains, although lacking complete PA biosynthetic pathways individually, can effectively cooperate metabolically, mimicking a modular biosynthetic system. The resulting PA concentrations, although moderate, suggest potential for further scale-up and process intensification in biotechnological applications.

The ability of *L. buchneri* A KKP 2047p to synthesize PDO was particularly noteworthy, as this strain lacks genes for both the methylglyoxal and lactate pathways, which are typically responsible for microbial PDO production. This raises the possibility that *L. buchneri* employs an alternative route, such as the deoxyhexose pathway, which has been described in bacteria utilizing rhamnose and fucose as substrates [13,16,41]. Further genomic and biochemical studies are required to confirm this hypothesis and to explore its implications for co-fermentation-based PA biosynthesis.

*L. buchneri* is known to metabolize PDO but grows poorly in media for which PDO is the sole carbon source [33,34]. Glucose supplementation has been reported to enhance bacterial growth and PDO utilization, leading to PA synthesis at levels of 174.9 mg/100 mL, which is consistent with the present study. Importantly, this process requires vitamin B12 supplementation, as *L. buchneri* lacks the genetic capacity for cobalamin biosynthesis. Vitamin B12 acts as an essential cofactor for propanediol dehydratase (EC 4.2.1.28), which catalyzes the dehydration of PDO to propionaldehyde—a key step in the PDO-to-PA conversion pathway. Without B12, this enzyme remains inactive, blocking PA biosynthesis through this route. This is unlike *L. reuteri*, which can produce PA in a modified MRS medium supplemented with PDO even in the absence of vitamin B_12_ [34]. These findings reinforce the importance of optimizing fermentation conditions to maximize LAB-driven PA production.

*C. maltaromaticum* IBB3447 represents a promising candidate for sustainable PDO and PA production due to its ability to metabolize glycerol, a major byproduct of biodiesel production. The utilization of glycerol by LAB is relatively rare, as most species lack the enzymatic capacity to convert glycerol into glycerone-phosphate, a key intermediate in glycolysis. However, certain *Carnobacterium* strains have been shown to possess this metabolic capability [39]. Notably, *C. maltaromaticum* IBB3447 efficiently converts glycerol into PDO, and in co-fermentation with complementary PA-producing strains, it contributes to PA biosynthesis, supporting the circular bioeconomy concept. This metabolic trait enhances its industrial potential, particularly in bioprocesses aimed at valorizing waste streams. The ability of *C. maltaromaticum* to utilize glycerol aligns with recent findings on polar *Carnobacterium* spp. that demonstrated efficient glycerol metabolism, suggesting that this feature could be more widespread within the genus [39]. Given the increasing need for bio-based chemical production and waste reduction, *C. maltaromaticum* IBB3447 could play a crucial role in sustainable PDO and PA fermentation, contributing to environmentally friendly biotechnological applications. Further research into its metabolic pathways and the optimization of fermentation conditions could enhance its efficiency in industrial bioprocessing, making it a valuable bio-based alternative for PDO and PA production from renewable resources.

*Propionibacterium* spp. remain the primary microbial producers of PA via the dicarboxylic acid pathway; however, their industrial application is limited by acid accumulation and pH-related growth inhibition [3,4]. Alternative pathways, such as the acrylate pathway in *Clostridium propionicum* and *Megasphaera elsdenii* and the PDO pathway in *Salmonella enterica*, suffer from metabolic inefficiencies or safety concerns due to the toxicity of metabolic intermediates or the virulence of the producing strains [5,6,7]. In contrast, LAB-driven PA biosynthesis offers a viable, sustainable alternative, using GRAS microorganisms with non-pathogenic metabolic pathways. Zielińska et al. [32] proposed a co-fermentation model involving *L. buchneri*, *L. diolivorans*, and *P. acidilactici*, suggesting that acetic acid synthesized by LAB could be converted into PDO, 1-propanol, and ultimately PA. While the exact metabolic routes remain unclear, field experiments have confirmed the presence of PDO and PA in plant material, demonstrating that these LAB strains can enhance PA concentrations in silage fermentations.

The present study confirms that carbon source selection significantly influences PA biosynthesis in LAB [34]. Among the tested sugars, mannose was the most potent inducer of antimicrobial compound production, likely due to its role in bacteriocin-mediated antagonistic activity. The mannose phosphotransferase system (Man-PTS) functions as a major receptor for class II bacteriocins [42]. The presence of mannose may lead to the overexpression of Man-PTS, increasing the receptor availability for bacteriocins and enhancing their antimicrobial activity. In contrast, this effect was not observed for glucose, fructose, or rhamnose, which do not induce similar Man-PTS-mediated bacteriocin interactions. These findings suggest a potential link between the sugar metabolism, transport systems, and antimicrobial properties of LAB, which may play a role in competitive microbial interactions within complex fermentation environments.

The study also evaluated PA biosynthesis in media containing alternative carbon sources beyond glucose, including polyalcohols (D-sorbitol, glycerol) and monosaccharides (fucose, rhamnose, fructose, sorbose, and mannose). Plant materials used in fermentation processes—such as grasses, alfalfa, energy crops, cucumbers, cabbage, and beets—contain various simple sugars that serve as microbial substrates [43,44]. Notably, sucrose, hydrolyzed into glucose and fructose, and inulin, a polymer of fructose found in Jerusalem artichoke, influence fermentation efficiency. Other plant-derived monosaccharides, including rhamnose, fucose, and sorbitol, also contribute to fermentation dynamics, highlighting their potential role in bioprocess optimization [45,46].

Beyond its significance for food preservation and silage fermentation, PA is a valuable industrial preservative, inhibiting microbial growth and enhancing product shelf life [18,19]. Additionally, there is growing industrial demand for PDO, a key precursor in PA biosynthesis, as a bio-based chemical in polyester resins and pharmaceuticals and as a food additive (E1520) [47,48] The shift toward biotechnological PDO production is driven by the high energy costs and environmental impact of petroleum-derived synthesis [49]. Experts predict that the global PDO market, valued at USD 335 million in 2020, will continue to expand, reinforcing the need for efficient, cost-effective microbial production strategies.

While this study provides valuable insights into LAB-driven PA biosynthesis, several limitations should be considered. First, although genomic analyses identified key genes related to PDO and PA metabolism, a functional validation through gene expression studies or knockout experiments was not performed. Additionally, while co-fermentation strategies demonstrated promising PA yields, industrial-scale feasibility was not assessed, and the further optimization of fermentation parameters is required to enhance production efficiency. The unexpected PDO synthesis in *L. buchneri* suggests the presence of alternative metabolic pathways, but their precise enzymatic mechanisms remain unknown and warrant further investigation. Finally, while glycerol utilization by *C. maltaromaticum* presents a promising approach for waste valorization, its efficiency compared to traditional PDO-producing strains needs to be further evaluated under industrial fermentation conditions.

## 5. Conclusions

This study demonstrates that LAB-driven co-fermentation optimizes PA production by enhancing PDO accumulation before its conversion into PA. The identification of C. maltaromaticum IBB3447 as a novel PDO producer, capable of utilizing glycerol for sustainable PDO synthesis, expands LAB’s metabolic potential and supports waste valorization in the circular bioeconomy. The unexpected PDO synthesis in *L. buchneri* despite lacking known pathways suggests the presence of alternative metabolic routes, warranting further investigation. The findings confirm that carbon source selection influences PA biosynthesis, with FRC-SOR-GLC yielding the highest PA concentrations. Given the rising demand for bio-based chemicals and natural preservatives, LAB fermentation presents a sustainable alternative to petrochemical PA and PDO production. Future research should optimize fermentation conditions, explore alternative PDO pathways, and scale-up LAB-based PA biosynthesis for industrial applications.

## Figures and Tables

**Table 1:** Antagonistic interactions between *C. maltaromaticum*, *L. buchneri*, and *L. brevis* strains under varying carbon sources and oxygen conditions. The inhibitory effects of different producers on indicator strains are presented under the following experimental conditions: glucose (GLC), rhamnose (RHA), fructose (FRC), or mannose (MAN) under microaerophilic (MICA), anaerobic (ANAR), or aerobic (AERO) conditions. The symbols indicate the degree of inhibition: filled circle—strong inhibition, open circle—moderate inhibition, and dashed open circle—no inhibition.

Indicators→	*C. maltaromaticum*						*L. buchneri*						*L. brevis*											
	IBB3437						A KKP 2047p						IBB3734						IBB3735					
Producers ↓	MICA/AERO/ANAR						MICA/AERO/ANAR						MICA/AERO/ANAR						MICA/AERO/ANAR					
	GLC			RHA	FRC	MAN	GLC			RHA	FRC	MAN	GLC			RHA	FRC	MAN	GLC			RHA	FRC	MAN
*C. maltaromaticum* IBB3447	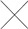	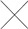	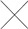	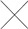	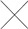	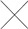	◌	◌	◌	◌	◌	●	◌	◌	◌	◌	◌	●	◌	◌	◌	◌	○	●
*L. buchneri* A KKP 2047p	◌	◌	◌	◌	◌	◌	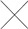	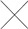	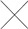	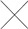	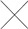	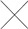	◌	◌	◌	◌	◌	○	◌	◌	◌	◌	◌	◌
*L. brevis* IBB3734	◌	◌	◌	◌	◌	◌	◌	◌	◌	◌	◌	◌	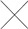	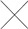	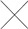	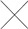	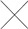	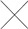	◌	◌	◌	◌	◌	◌
*L. brevis* IBB3735	◌	◌	◌	◌	◌	◌	◌	◌	◌	◌	◌	○	◌	◌	◌	◌	◌	○	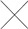	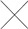	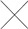	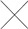	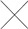	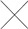

**Table 2:** Production of PDO and PA in co-culture fermentation using the stepwise approach. PDO—1,2-propanediol, PA—propionic acid, GLC—glucose, FRC—fructose, RHA—rhamnose, SOR—sorbitol.

Monoculture					Coculture				
No. 1 Strain	Carbon Source	Time	PDO [mM]	PA [mM]	No. 2 Strain	Carbon Source	Time	PDO [mM]	PA [mM]
*C. maltaromaticum* IBB3447	GLY	96 h	1.36	0	*L. brevis* IBB3735	GLC	96 h	0	0.73
*C. maltaromaticum* IBB3447	GLY	96 h	1.20	0	*L. brevis* IBB3734	GLC	96 h	0	0.60
*C. maltaromaticum* IBB3447	SOR	96 h	1.52	0	*L. brevis* IBB3735	GLC	96 h	0	0.19
*C. maltaromaticum* IBB3447	SOR	96 h	1.39	0	*L. brevis* IBB3734	GLC	96 h	0.01	0.17
*C. maltaromaticum* IBB3447	RHA	96 h	1.41	0	*L. brevis* IBB3735	GLC	96 h	0	0.29
*C. maltaromaticum* IBB3447	RHA	96 h	1.30	0	*L. brevis* IBB3734	GLC	96 h	0	0.21
*L. buchneri* A KKP 2047p	FRC	14 days	3.10	0	*L. brevis* IBB3735	GLC	96 h	1.81	3.31
*L. buchneri* A KKP 2047p	FRC	14 days	3.65	0	*L. brevis* IBB3734	GLC	96 h	1.57	1.45

**Table 3:** Production of PDO and PA in co-culture fermentation using the simultaneous fermentation approach. PDO—1,2-propanediol, PA—propionic acid, GLC—glucose, FRC—fructose, RHA—rhamnose, SOR—sorbitol.

Strains in Co-Culture	Carbon Source	Time	PDO in Co-Culture [mM]	PA in Co-Culture [mM]
*C. maltaromaticum* IBB3447	GLC-SOR	72 h	0.92	0.06
*L. brevis* IBB3735		96 h	0.71	0.19
		7 days	0.31	0.60
		14 days	0.02	0.92
*C. maltaromaticum* IBB3447	GLC-RHA	72 h	0.88	0.02
*L. brevis* IBB3735		96 h	0.59	0.11
		7 days	0.11	0.41
		14 days	0	0.78
*C. maltaromaticum* IBB3447	FRC-SOR	72 h	0.22	0
*L. buchneri* A KKP 2047p		96 h	0.86	0
		7 days	2.14	0
		14 days	12.11	0
*C. maltaromaticum* IBB3447	FRC-RHA	72 h	0.36	0
*L. buchneri* A KKP 2047p		96 h	0.63	0
		7 days	4.8	0
		14 days	13.13	0
*C. maltaromaticum* IBB3447	FRC-SOR-GLC	72 h	5.11	1.13
*L. buchneri* A KKP 2047p		96 h	6.01	4.45
*L. brevis* IBB3735		7 days	1.59	6.66
		14 days	0.41	7.09
*C. maltaromaticum* IBB3447	FRC-RHA-GLC	72 h	7.17	0.99
*L. buchneri* A KKP 2047p		96 h	5.48	5.58
*L. brevis* IBB3735		7 days	0.9	5.94
		14 days	0.22	6.87

## Data Availability

Sequencing data have been deposited in GenBank under BioProject PRJNA1232960 (BioSample SAMN47255224) for *Levilactobacillus brevis* IBB3734 (accession number JBMAFC000000000); BioProject PRJNA1232964 (BioSample SAMN47255257) for *Levilactobacillus brevis* IBB3735 (accession number JBMAFD000000000); BioProject PRJNA1232980 (BioSample SAMN47256067) for *Lentilactobacillus buchneri* A KKP 2047p (accession number JBMAFE000000000); and BioProject PRJNA1233225 (BioSample SAMN47263590) for *Carnobacterium maltaromaticum* IBB3447 (accession number JBMAFF000000000). Illumina raw sequencing reads are available in the SRA database under accession no. SRR32632078, SRR32630978, SRR32630301, and SRR32630234 for, respectively, *L. buchneri* A KKP 2047p, *L. brevis* IBB3735, *L. brevis* IBB3734, and *C. maltaromaticum* IBB3447.

## Supplementary Materials

The following supporting information can be downloaded at: https://www.mdpi.com/article/10.3390/foods14091573/s1, Figure S1: Distribution of enzymes involved in the conversion of intermediates to PA. The intensity of green coloration in the pathway diagram highlights the most widely distributed enzymes. The intermediates of the glycerone-P-propionyl-CoA conversion pathway are indicated by red circles; Figure S2: Distribution of enzymes involved in the conversion of glycerone-P to propionyl-CoA within the group I (PDO producers) and group II (PA producers) bacteria. (A) *L. buchneri* A KKP 2047p, (B) *C. maltaromaticum* IBB3447, (C) *L. brevis* IBB3734, (D) *L. brevis* IBB3735. Green indicates the presence of the gene encoding the enzyme in the genome; white, none; Figure S3: Relative PDO content in medium supplemented with different carbon sources for *C. maltaromaticum* IBB 3447. The highest PDO concentration was observed in the D-sorbitol variant after 96 h; Figure S4: Relative PDO content in medium supplemented with different carbon sources for *L. buchneri* A KKP 2047. The highest PDO concentration was observed in the fructose variant after 14 days; Figure S5: Relative PA content in medium supplemented with PDO and different carbon sources for *L. brevis* IBB3734 (a) and IBB3735 (b). The highest PA concentration was observed in the glucose variant after 96 h for both *L. brevis* strains.

## Abbreviations

The following abbreviations are used in this manuscript:

PA	propionic acid
LAB	lactic acid bacteria
PDO	1,2-propanediol
