# Supplementary material for: Co-Fermentation and Genomic Insights into Lactic Acid Bacteria for Enhanced Propionic Acid Production Using a Non-GMO Approach
Source: Foods. 2025 Apr 29;14(9):1573. doi: 10.3390/foods14091573 (PMC12071468; doi:10.3390/foods14091573)

Supplementary materials

Co-fermentation and genomic insights into lactic acid bacteria for enhanced propionic acid production in a non-GMO approach

Lidia Stasiak-Różańska[1], Jan Gawor[2], Kamil Piwowarek[3] Agata Fabiszewska[4] and Tamara Aleksandrzak-Piekarczyk[2,*]

[1]Department of Food Technology and Assessment, Institute of Food Sciences, Warsaw University of Life Sciences, Nowoursynowska St. 159c, 02-776, Warsaw, Poland; lidia_stasiak_rozanska@sggw.edu.pl
[2] Institute of Biochemistry and Biophysics, Polish Academy of Sciences, Pawinskiego 5a, 02-106 Warsaw, Poland; gaworj@ibb.waw.pl; tamara@ibb.waw.pl
[3] Department of Food Biotechnology and Microbiology, Institute of Food Sciences, Warsaw University of Life Sciences, Nowoursynowska St. 159c, 02-776, Warsaw, Poland; kamil_piwowarek@sggw.edu.pl
[4] Department of Chemistry, Institute of Food Sciences, Warsaw University of Life Sciences, Nowoursynowska St. 159c, 02-776, Warsaw, Poland; agata_fabiszewska@sggw.edu.pl
*Corresponding author. Mailing address: IBB PAS, Pawińskiego 5a, 02-106 Warsaw, Poland, E-mail: tamara@ibb.waw.pl

Table S1. Statistics of the de novo assemblies of four strains sequenced in this study

Organism	*L. brevis*		*L. buchneri*	*C. maltaromaticum*
	**IBB3734**	**IBB3735**	**KKP 2047p**	**IBB3447**
accession no	NZ_JBMAFC000000000	NZ_JBMAFD000000000	NZ_JBMAFE000000000	NZ_JBMAFF000000000
Assembly:	GCF_048565605.1	GCF_048565585.1	GCF_048565685.1	GCF_048567425.1
Genome size (bp)	2,550,651	2,510,261	2,447,364	3,479,940
GC content (%)	45.5	45.5	44.5	34.5
CDS	2,558	2,538	2,335	3,184
Contigs	120	134	83	33
Genome coverage (Illumina)	225x	214x	197x	96x

Figure S1. Distribution of enzymes involved in the conversion of intermediates to PA. The intensity of green coloration in the pathway diagram highlights the most widely distributed enzymes. The intermediates of the glycerone-P-propionyl-CoA conversion pathway are indicated by red circles.

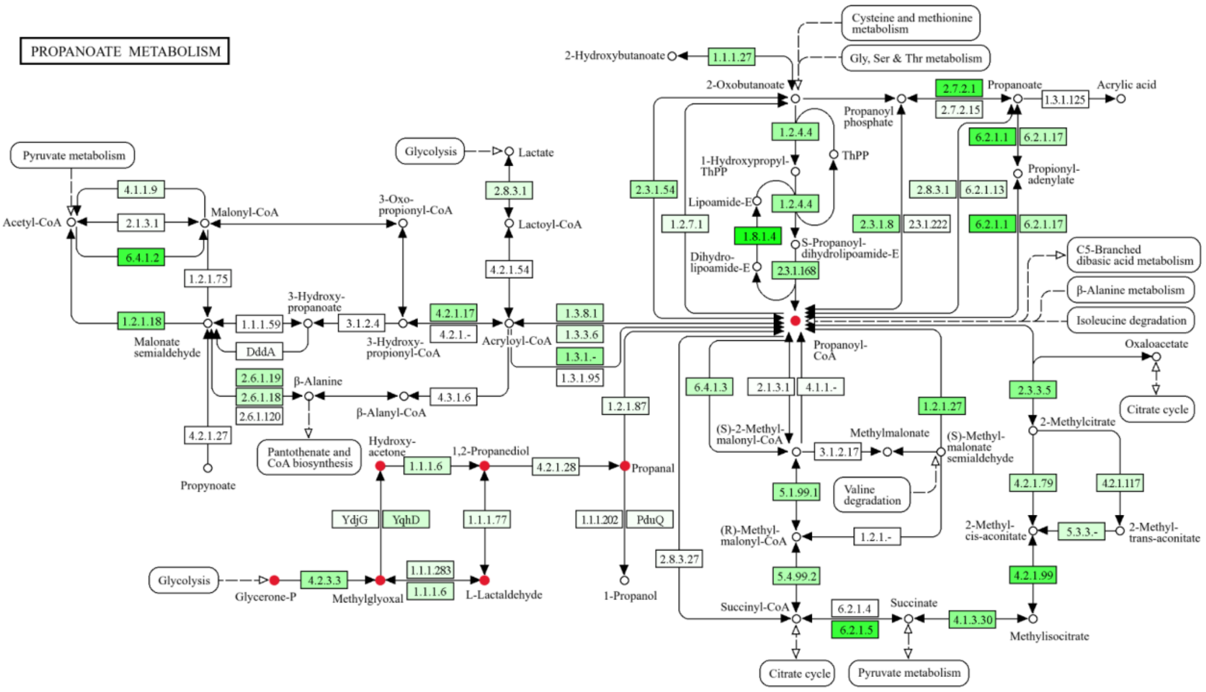

Figure S2. Distribution of enzymes involved in the conversion of glycerone-P to propionyl-CoA within the group I (PDO producers) and group II (PA producers) bacteria. (A) *L. buchneri* A KKP 2047p, (B) *C. maltaromaticum* IBB3447, (C) *L. brevis* IBB3734, (D) *L. brevis* IBB3735. Green indicates the presence of the gene encoding the enzyme in the genome; white, none.

A

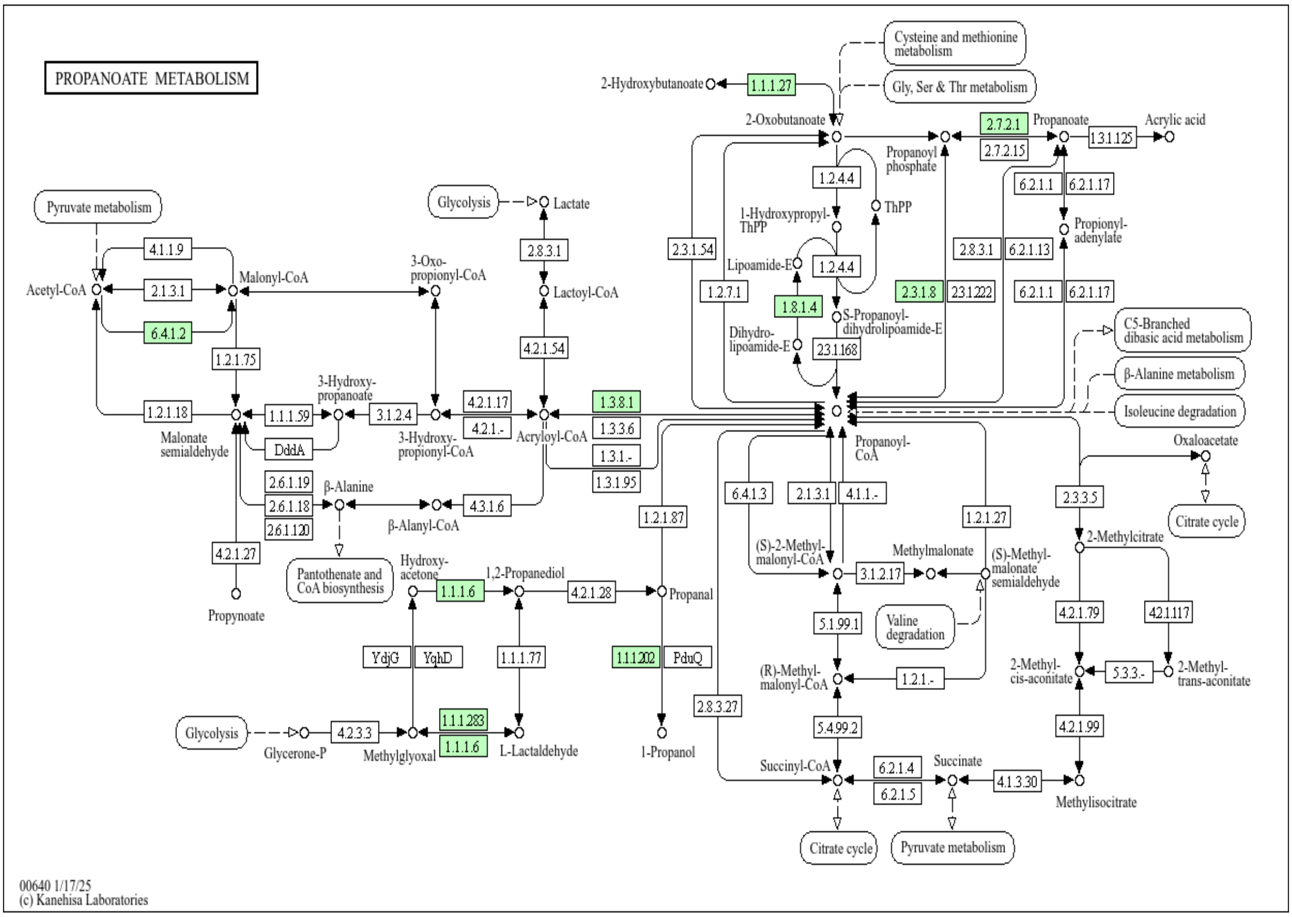

B

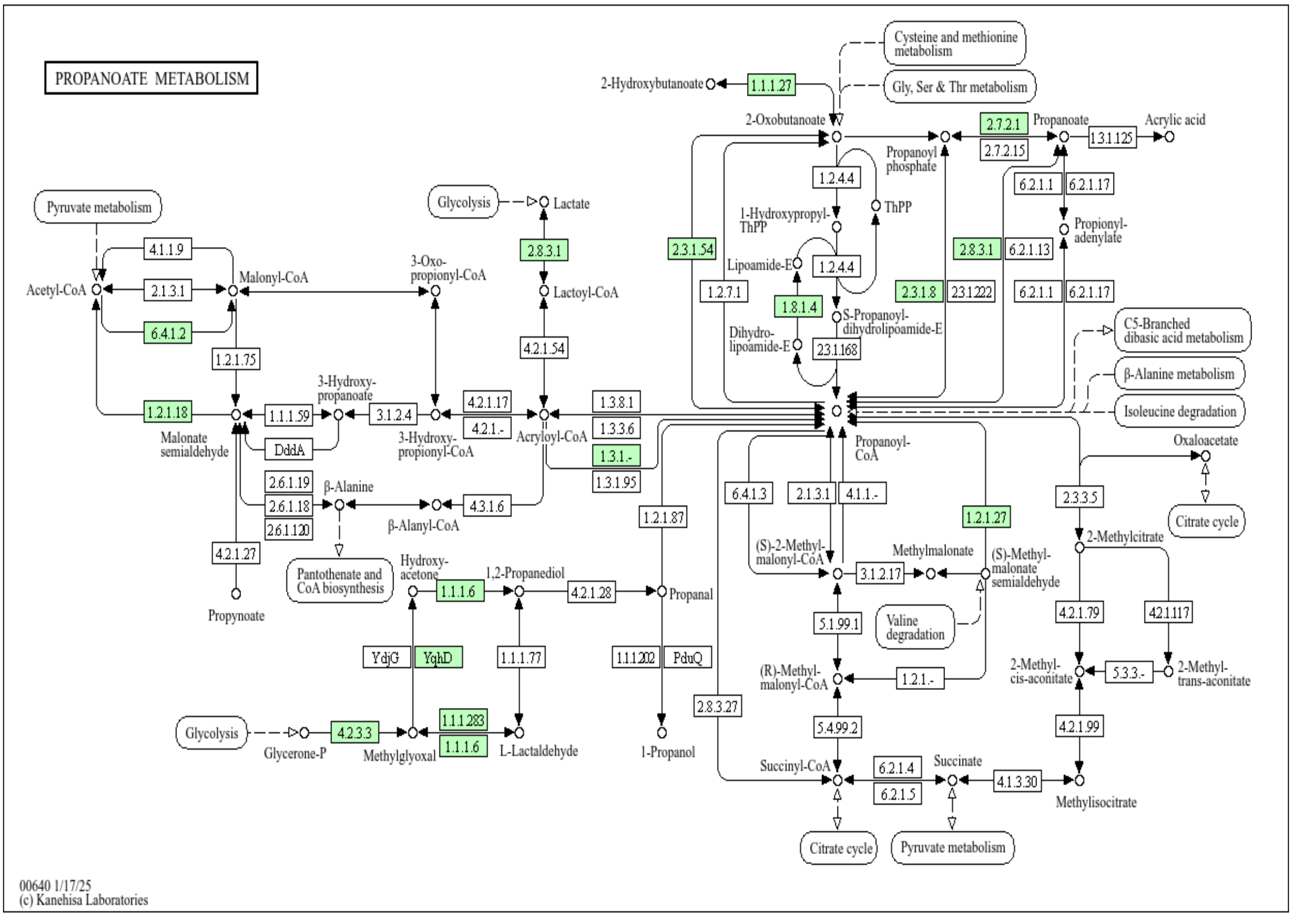

PROPANOATE METABOLISM
Cysteine and methionine metabolism
Gly, Ser & Thr metabolism
2-Hydroxybutanoate
1.1.1.27
2-Oxobutanoate
2.7.2.1
2.7.2.15
Propanoate
1.3.1.125
Acrylic acid
Propanoyl phosphate
1.2.4.4
1-Hydroxypropyl-ThPP
ThPP
Lipoamide-E
Dihydro-lipoamide-E
S-Propanoyl-dihydrolipoamide-E
1.8.1.4
2.3.1.168
2.3.1.54
1.2.7.1
2.8.3.1
2.3.1.8
2.3.1.222
6.2.1.1
6.2.1.17
6.2.1.13
Propionyl-adenylate
C5-Branched dibasic acid metabolism
β-Alanine metabolism
Isoleucine degradation
Pyruvate metabolism
Glycolysis
Lactate
Lactoyl-CoA
4.2.1.54
Acetyl-CoA
Malonyl-CoA
4.1.1.9
2.1.3.1
6.4.1.2
3-Oxo-propionyl-CoA
1.2.1.75
1.2.1.18
Malonate semialdehyde
1.1.1.59
3-Hydroxy-propanoate
3.1.2.4
3-Hydroxy-propionyl-CoA
4.2.1.17
4.2.1.-
Acryloyl-CoA
1.3.8.1
1.3.3.6
1.3.1.-
1.3.1.95
DddA
2.6.1.19
2.6.1.18
2.6.1.120
β-Alanine
4.3.1.6
β-Alanyl-CoA
4.2.1.27
Propynoate
Pantothenate and CoA biosynthesis
Hydroxy-acetone
1.1.1.6
1,2-Propanediol
4.2.1.28
Propanal
1.2.1.87
YdjG
YqhD
1.1.1.77
1.1.1.202
PduQ
1-Propanol
Glycerone-P
4.2.3.3
Methylglyoxal
1.1.1.283
L-Lactaldehyde
2.8.3.27
Propanoyl-CoA
6.4.1.3
4.1.1.-
1.2.1.27
(S)-2-Methyl-malonyl-CoA
3.1.2.17
Methylmalonate
(S)-Methyl-malonate semialdehyde
Valine degradation
5.1.99.1
(R)-Methyl-malonyl-CoA
1.2.1.-
5.4.99.2
Succinyl-CoA
6.2.1.4
6.2.1.5
Succinate
4.1.3.30
Methylisocitrate
Citrate cycle
Oxaloacetate
2.3.3.5
2-Methylcitrate
4.2.1.79
4.2.1.117
2-Methyl-cis-aconitate
5.3.3.-
2-Methyl-trans-aconitate
4.2.1.99
00640 1/17/25
(c) Kanehisa Laboratories

C

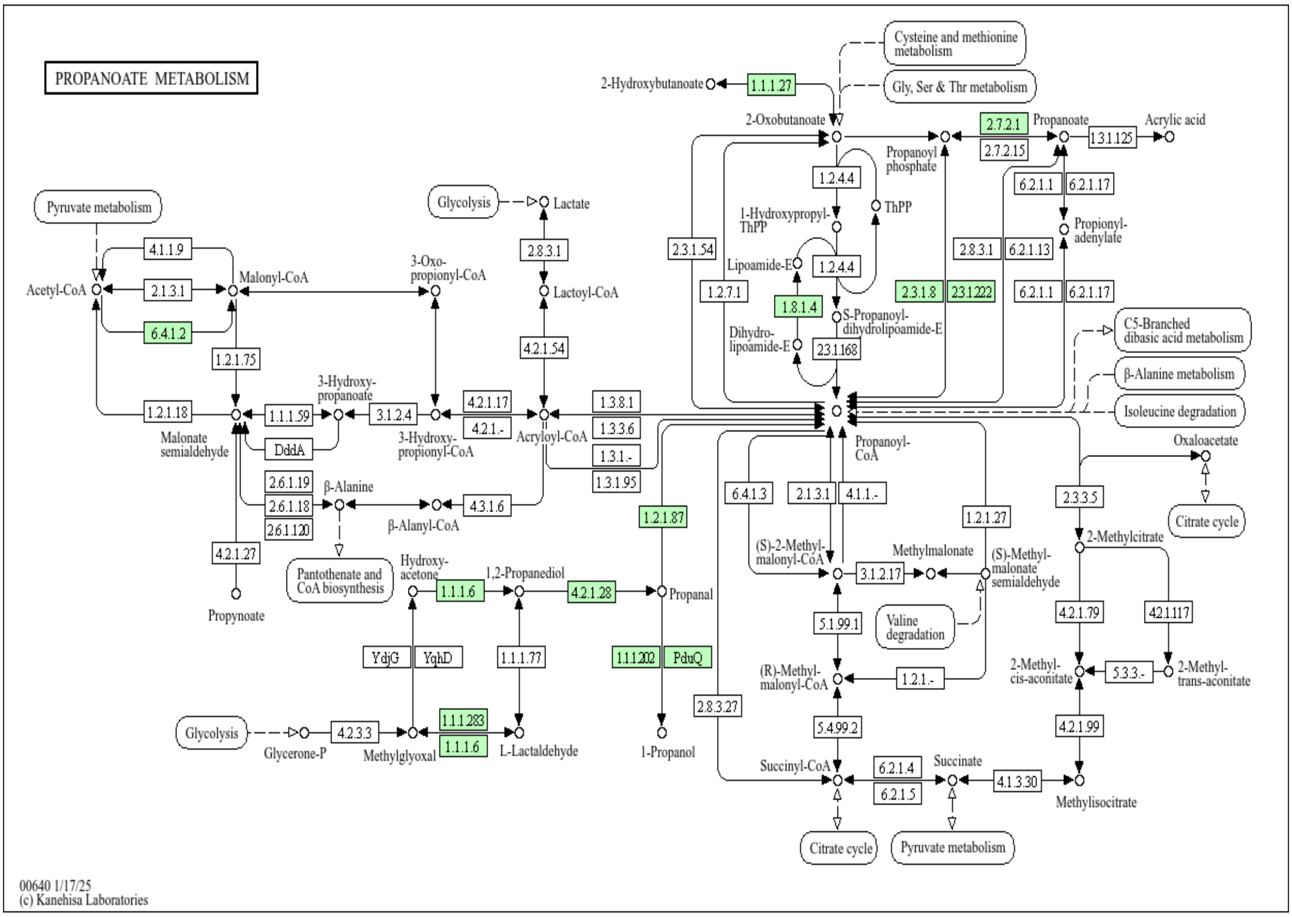

PROPANOATE METABOLISM
Cysteine and methionine metabolism
Gly, Ser & Thr metabolism
2-Hydroxybutanoate
1.1.1.27
2-Oxobutanoate
2.7.2.1
2.7.2.15
Propanoate
1.3.1.125
Acrylic acid
Propanoyl phosphate
1.2.4.4
ThPP
1-Hydroxypropyl-ThPP
6.2.1.1
6.2.1.17
Propionyl-adenylate
Pyruvate metabolism
Glycolysis
Lactate
2.8.3.1
4.1.1.9
2.1.3.1
6.4.1.2
Acetyl-CoA
Malonyl-CoA
3-Oxo-propionyl-CoA
Lactoyl-CoA
2.3.1.54
Lipoamide-E
1.2.7.1
1.8.1.4
2.3.1.8
2.3.1.222
6.2.1.13
S-Propanoyl-dihydrolipoamide-E
Dihydro-lipoamide-E
2.3.1.168
C5-Branched dibasic acid metabolism
β-Alanine metabolism
Isoleucine degradation
1.2.1.75
4.2.1.54
3-Hydroxy-propanoate
4.2.1.17
4.2.1.-
1.3.8.1
1.3.3.6
1.3.1.-
1.3.1.95
1.2.1.18
1.1.1.59
3.1.2.4
Malonate semialdehyde
DddA
3-Hydroxy-propionyl-CoA
Acryloyl-CoA
Propanoyl-CoA
Oxaloacetate
2.6.1.19
2.6.1.18
2.6.1.120
β-Alanine
4.3.1.6
β-Alanyl-CoA
1.2.1.87
6.4.1.3
2.1.3.1
4.1.1.-
1.2.1.27
2.3.3.5
Citrate cycle
2-Methylcitrate
4.2.1.27
Pantothenate and CoA biosynthesis
Hydroxy-acetone
1,2-Propanediol
1.1.1.6
4.2.1.28
Propanal
(S)-2-Methyl-malonyl-CoA
3.1.2.17
Methylmalonate
(S)-Methyl-malonate semialdehyde
Propynoate
YdjG
YqhD
1.1.1.77
1.1.1.202
PduQ
5.1.99.1
Valine degradation
4.2.1.79
4.2.1.117
2-Methyl-cis-aconitate
5.3.3.-
2-Methyl-trans-aconitate
(R)-Methyl-malonyl-CoA
1.2.1.-
2.8.3.27
1.1.1.283
Glycolysis
4.2.3.3
Glycerone-P
Methylglyoxal
L-Lactaldehyde
1-Propanol
5.4.99.2
Succinyl-CoA
6.2.1.4
6.2.1.5
Succinate
4.1.3.30
4.2.1.99
Methylisocitrate
Citrate cycle
Pyruvate metabolism
00640 1/17/25
(c) Kanehisa Laboratories

D

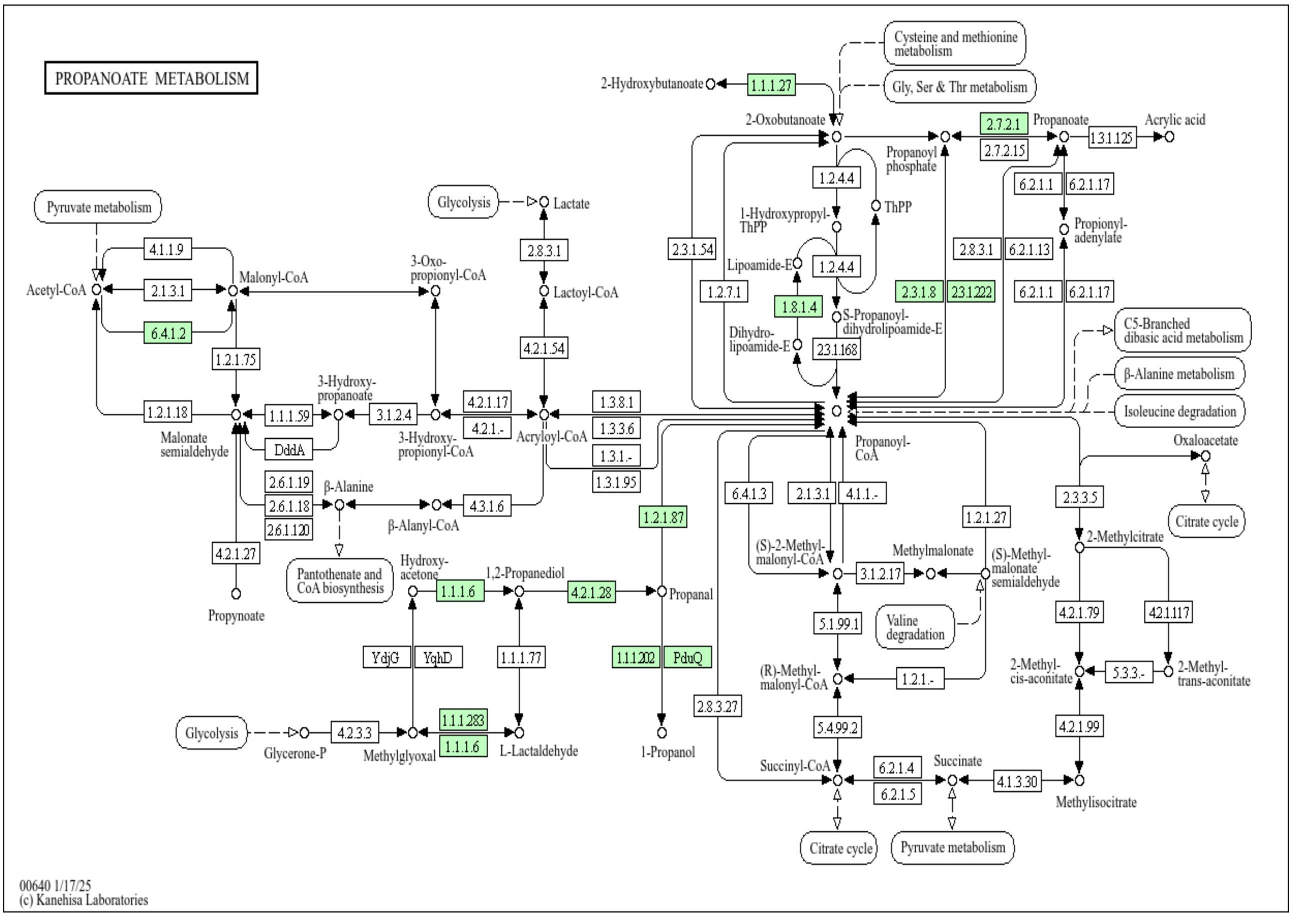
PROPANOATE METABOLISM
Cysteine and methionine metabolism
Gly, Ser & Thr metabolism
2-Hydroxybutanoate
1.1.1.27
2-Oxobutanoate
2.7.2.1
2.7.2.15
Propanoate
1.3.1.125
Acrylic acid
Propanoyl phosphate
1.2.4.4
1-Hydroxypropyl-ThPP
ThPP
Lipoamide-E
1.8.1.4
S-Propanoyl-dihydrolipoamide-E
Dihydro-lipoamide-E
2.3.1.168
2.3.1.54
1.2.7.1
2.8.3.1
2.3.1.8
2.3.1.222
6.2.1.1
6.2.1.17
6.2.1.13
Propionyl-adenylate
C5-Branched dibasic acid metabolism
β-Alanine metabolism
Isoleucine degradation
Pyruvate metabolism
Glycolysis
Lactate
2.8.3.1
Lactoyl-CoA
4.2.1.54
Acetyl-CoA
4.1.1.9
2.1.3.1
6.4.1.2
Malonyl-CoA
3-Oxo-propionyl-CoA
1.2.1.75
1.2.1.18
Malonate semialdehyde
1.1.1.59
DddA
3-Hydroxy-propanoate
3.1.2.4
3-Hydroxy-propionyl-CoA
4.2.1.17
4.2.1.-
Acryloyl-CoA
1.3.8.1
1.3.3.6
1.3.1.-
1.3.1.95
Propanoyl-CoA
2.6.1.19
2.6.1.18
2.6.1.120
β-Alanine
4.3.1.6
β-Alanyl-CoA
4.2.1.27
Propynoate
Pantothenate and CoA biosynthesis
Hydroxy-acetone
1.1.1.6
1,2-Propanediol
4.2.1.28
Propanal
1.2.1.87
YdjG
YqhD
1.1.1.77
1.1.1.202
PduQ
1-Propanol
Glycolysis
Glycerone-P
4.2.3.3
Methylglyoxal
1.1.1.283
1.1.1.6
L-Lactaldehyde
6.4.1.3
2.1.3.1
4.1.1.-
(S)-2-Methyl-malonyl-CoA
3.1.2.17
Methylmalonate
1.2.1.27
(S)-Methyl-malonate semialdehyde
Valine degradation
5.1.99.1
(R)-Methyl-malonyl-CoA
1.2.1.-
5.4.99.2
2.8.3.27
Succinyl-CoA
6.2.1.4
6.2.1.5
Succinate
4.1.3.30
Methylisocitrate
Citrate cycle
Pyruvate metabolism
Oxaloacetate
Citrate cycle
2.3.3.5
2-Methylcitrate
4.2.1.79
4.2.1.117
2-Methyl-cis-aconitate
5.3.3.-
2-Methyl-trans-aconitate
4.2.1.99
00640 1/17/25
(c) Kanehisa Laboratories

Figure S3. Relative PDO content in medium supplemented with different carbon sources for *C. maltaromaticum* IBB 3447. The highest PDO concentration was observed in the D-sorbitol variant after 96 h.

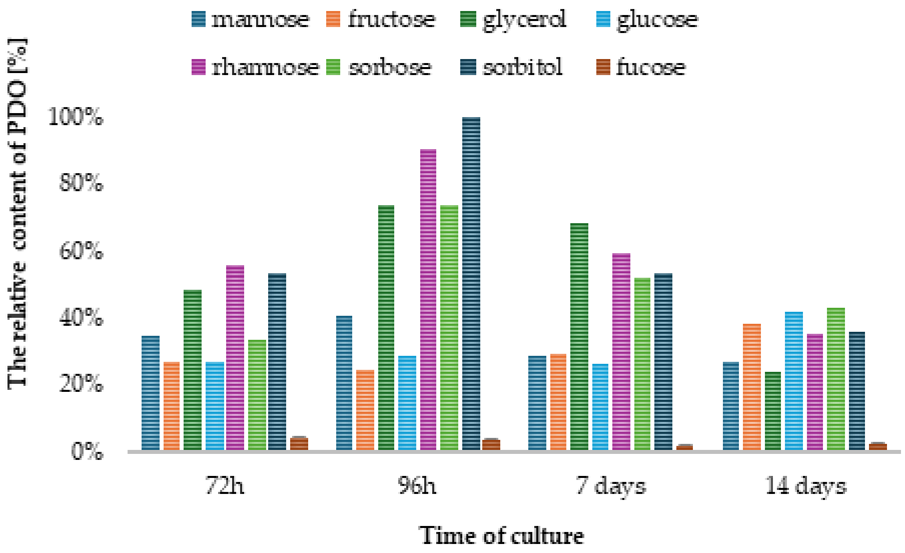

Figure S4. Relative PDO content in medium supplemented with different carbon sources for *L. buchneri* A KKP 2047. The highest PDO concentration was observed in the fructose variant after 14 days.

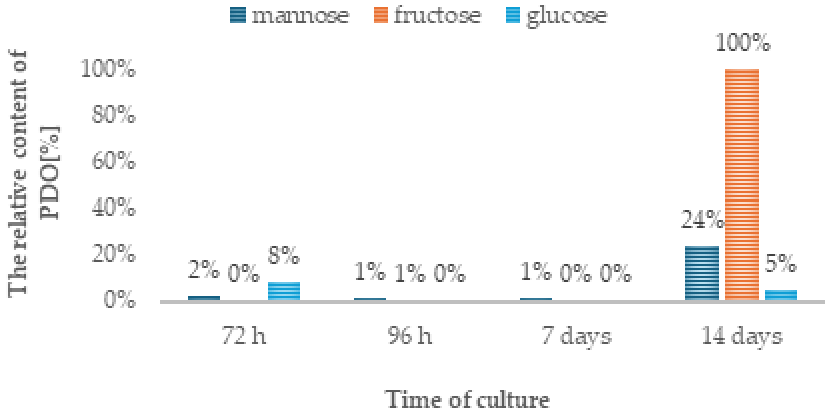

Figure S5. Relative PA content in medium supplemented with PDO and different carbon sources for *L. brevis* IBB3734 (a) and IBB3735 (b). The highest PA concentration was observed in the glucose variant after 96 h for both *L. brevis* strains.

a)

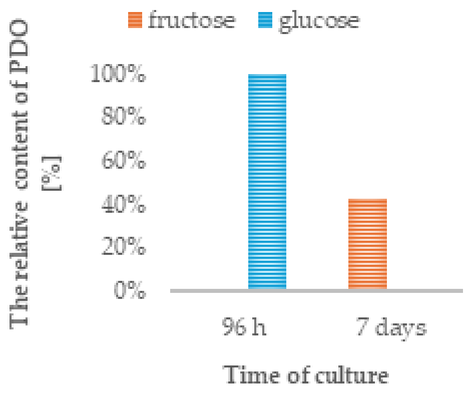

b)

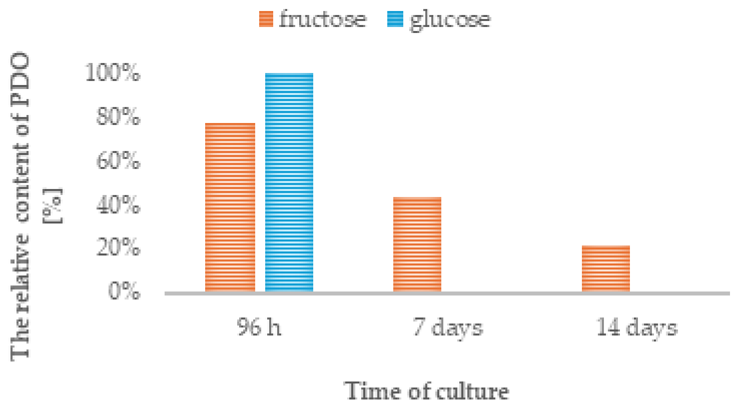

Supplement: Supplementary file 1 [file foods-14-01573-s001.zip › foods-3563067-supplementary.pdf]
